# Efficacy of attention bias modification training for depressed adults: a randomized clinical trial

**DOI:** 10.1017/S0033291721000702

**Published:** 2022-12

**Authors:** Kean J. Hsu, Jason Shumake, Kayla Caffey, Semeon Risom, Jocelyn Labrada, Jasper A. J. Smits, David M. Schnyer, Christopher G. Beevers

**Affiliations:** 1 Georgetown University Medical Center, Washington, DC, USA; 2 Institute for Mental Health Research and Department of Psychology, University of Texas at Austin, Austin, TX, USA

**Keywords:** Attention bias modification training, depression, negative attentional bias, randomized clinical trial

## Abstract

**Background:**

This study examined the efficacy of attention bias modification training (ABMT) for the treatment of depression.

**Methods:**

In this randomized clinical trial, 145 adults (77% female, 62% white) with at least moderate depression severity [i.e. self-reported Quick Inventory of Depressive Symptomatology (QIDS-SR) ⩾13] and a negative attention bias were randomized to active ABMT, sham ABMT, or assessments only. The training consisted of two in-clinic and three (brief) at-home ABMT sessions per week for 4 weeks (2224 training trials total). The pre-registered primary outcome was change in QIDS-SR. Secondary outcomes were the 17-item Hamilton Depression Rating Scale (HRSD) and anhedonic depression and anxious arousal from the Mood and Anxiety Symptom Questionnaire (MASQ). Primary and secondary outcomes were administered at baseline and four weekly assessments during ABMT.

**Results:**

Intent-to-treat analyses indicated that, relative to assessment-only, active ABMT significantly reduced QIDS-SR and HRSD scores by an additional 0.62 ± 0.23 (*p* = 0.008, *d* = −0.57) and 0.74 ± 0.31 (*p* = 0.021, *d* = −0.49) points per week. Similar results were observed for active *v.* sham ABMT: a greater symptom reduction of 0.44 ± 0.24 QIDS-SR (*p* = 0.067, *d* = −0.41) and 0.69 ± 0.32 HRSD (*p* = 0.033, *d* = −0.42) points per week. Sham ABMT did not significantly differ from the assessment-only condition. No significant differences were observed for the MASQ scales.

**Conclusion:**

Depressed individuals with at least modest negative attentional bias benefitted from active ABMT.

## Introduction

Cognitive models of depression posit that a negative attentional bias may play an important role in the maintenance of depression symptoms and major depressive disorder (MDD; Disner, Beevers, Haigh, & Beck, 2011; Joormann & Vanderlind, 2014). Specifically, these models suggest that depressed individuals preferentially attend to and have difficulty in disengaging from sad stimuli. This attentional bias is hypothesized to lead to persistent sad mood and maintain depression (Disner et al., 2011).

Empirical research has supported these assertions. Meta-analyses report that depressed adults exhibit biased attention for negative stimuli and preferential gaze for sad stimuli compared to healthy control individuals (Armstrong & Olatunji, 2012; Peckham, McHugh, & Otto, 2010; Suslow, Hußlack, Kersting, & Bodenschatz, 2020). These biases in depression likely reflect difficulties in disengaging from sad stimuli (e.g. Sanchez, Vazquez, Marker, LeMoult, & Joormann, 2013) and relatively late-stage attentional processes (1000 ms onward; De Raedt & Koster, 2010). Negative attentional biases are also associated with the persistence of sad mood and impaired mood recovery in adults diagnosed with depression (Clasen, Wells, Ellis, & Beevers, 2013; Sanchez et al., 2013). Finally, negative attentional bias is associated with symptom worsening across 1 month among individuals with elevated symptoms of depression (Disner, Shumake, & Beevers, 2017).

Experimental studies that modify negative attentional bias (i.e. attention bias modification training; ABMT) in depressed individuals provide a strong test of whether preferential attention to negative information maintains depression. In most of these studies, tasks are constructed to repeatedly direct attention away from negative stimuli and toward neutral or positive stimuli. With repeated practice, the depressed person putatively learns to overcome the habitual tendency to focus on negative information and instead attend to non-negative stimuli. Individuals receiving ABMT often show significant reductions in depression symptoms from pre- to post-training; however, in some cases active ABMT did not outperform sham ABMT (Beevers, Clasen, Enock, & Schnyer, 2015; Mastikhina & Dobson, 2017; Wells & Beevers, 2010; Yang, Ding, Dai, Peng, & Zhang, 2015).

One explanation for these mixed findings may be that not all depressed individuals have a negative attentional bias. Indeed, recent research suggests that a negative attentional bias may be required for successful active ABMT, although large samples are required to accurately test whether pre-training bias moderates the efficacy of ABMT (Kruijt & Carlbring, 2018; Wang & Ware, 2013). A complementary approach is to recruit depressed adults with a negative attention bias and determine whether ABMT is effective in this subgroup.

The aim of the current study was to test the efficacy of active ABMT and sham ABMT compared to assessments only in a sample of adults with elevated depression severity and at least a moderate negative attentional bias. We hypothesized that active ABMT would lead to significantly greater reductions in depression symptoms compared to the completion of assessments only (Hsu et al., 2018). We did not have a strong hypothesis about the effects of sham ABMT on symptoms relative to active ABMT (Hsu et al., 2018) – sham ABMT has reduced depression symptoms in some studies (e.g. Beevers et al., 2015) but not in others (e.g. Yang et al., 2015), and has been studied as a treatment for post-traumatic stress disorder under the label of ‘attention control training’ (Badura-Brack et al., 2015). These findings suggest that sham ABMT may not be an inert comparison condition. Although sham ABMT provides important mechanistic evidence about the necessity of targeting a negative attentional bias, if both sham and active ABMT lead to symptom reduction it will be impossible to determine whether symptom change is due to non-specific factors (e.g. the passage of time or interaction with study staff) or the therapeutic properties of ABMT. Thus, as documented in our pre-registered study protocol (Hsu et al., 2018), we included an assessment-only comparison condition to provide important information about the *efficacy* of active and sham ABMT for depression.

## Methods

### Participants

**Fig. 1.**
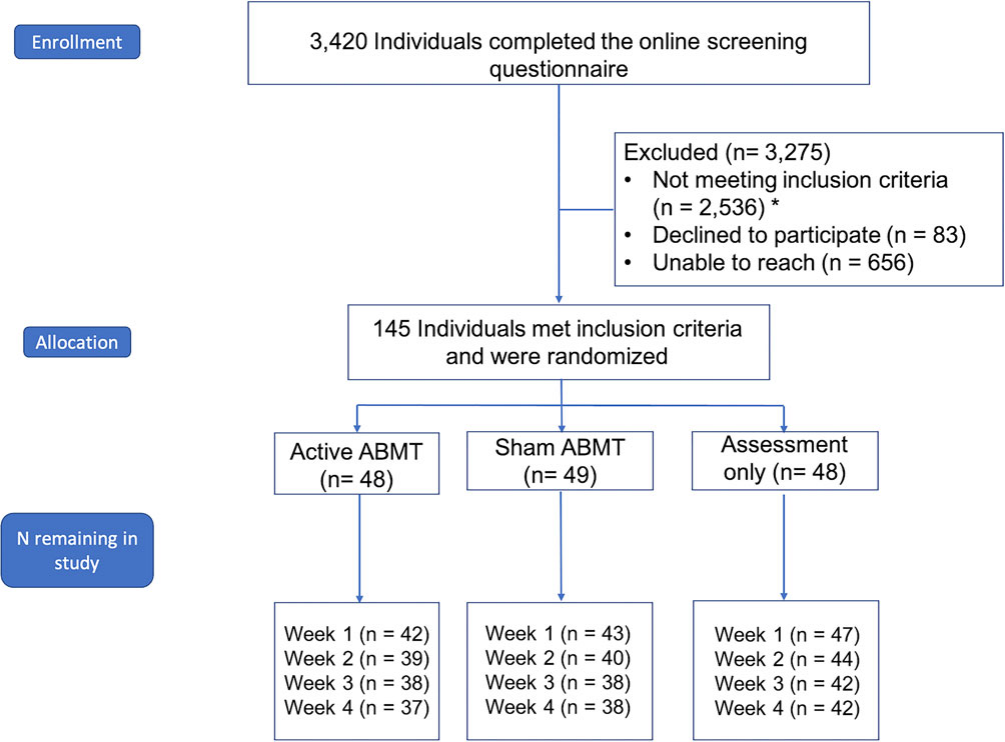
Consort diagram. CONSORT diagram documenting participant flow through the study. All available data were analyzed. *The same person may contribute to the count of more than one exclusionary criterion; in addition, although we required individuals to be stable on antidepressant medications for at least 12 weeks without a change in dosage or medication, we actually did not exclude anyone due to this criterion because: (1) after they were stable on their medication for 12 weeks they were re-evaluated for inclusion/exclusion criteria and they were excluded for another reason; (2) after they were stable on their medication for 12 weeks they were re-evaluated for inclusion/exclusion criteria and they were eligible and enrolled; or (3) we were unable to recontact participants after the 12 week waiting period and they were excluded for that reason.

Participants in this single center, randomized, blinded clinical trial (NCT 02880215) were 145 adults recruited from the Austin, TX, USA community through social media advertisements, flyering, and community postings among other approaches. Sample size was determined *a priori* by a power analysis to detect a significant difference in the rate of symptom change between the active ABMT and assessment-only condition (see study protocol; Hsu et al., 2018). Based on prior research (Beevers et al., 2015; Disner et al., 2017), results from the power analysis indicated requiring 111 completers to detect a significant training group × time interaction. We initially sought to recruit 123 participants based on an assumption of 10% attrition by study end. We monitored attrition throughout the study and given that attrition was higher (19.3%) than expected, we recruited as many additional participants beyond *n* = 123 as pragmatically possible to maximize statistical power (see Fig. 1 for CONSORT diagram). No analyses were performed until recruitment was complete. More details about analyses, data location, and analysis code can be found in online Supplementary Appendix 1 (SA1). Subjects provided written informed consent after receiving a complete description of the study.

#### Inclusion criteria

Participants were eligible if they could provide informed consent, were fluent in English, scored 13 or greater on the Quick Inventory of Depressive Symptoms-Self-Report (QIDS-SR) at the baseline assessment, and had at least a modest attentional bias for dysphoric stimuli relative to neutral stimuli on a dot-probe task (see section ‘Assessments’). Based on pilot data indicating that 1 week test–retest reliability was *r* = 0.67, a modest negative attentional bias was defined as having at least 37.5% of trials (36 out of 96) where summed eye gaze dwell time was greater for sad stimuli than neutral stimuli on a dot-probe task (for further details, see Hsu et al., 2018). We also required participants to be between 18 and 40 years of age to minimize the impact of cognitive aging on outcomes to be examined in forthcoming reports [e.g. functional connectivity during resting-state, functional magnetic resonance imaging (fMRI)].

#### Exclusion criteria

Participants were excluded if they reported suicidal behavior or significant suicidal ideation within the past 6 months using the Columbia-Suicide Severity Rating Scale (C-SSRS; Posner et al., 2011), if they met criteria for current or past bipolar or psychotic disorders or for substance use disorders of mild or greater severity within the past 12 months on the Mini International Neuropsychiatric Interview (MINI; Lecrubier, Sheehan, Hergueta, & Weiller, 1998), or if they were taking current opioid analgesics or systemic corticosteroid use as these medications could impact neuroimaging data collection. Individuals on antidepressant medication were required to have had no changes in medication and dosage in the past 12 weeks.

### Assessments

All primary, secondary, and treatment target (i.e. attention bias) assessments were obtained at baseline and then weekly for 4 weeks (i.e. week 0, 1, 2, 3, 4) for a total of five assessments. Detailed information about study assessments can be found in the published study protocol (Hsu et al., 2018). Inter-rater agreement (Fleiss' kappa) for current MDD, lifetime MDD, and recurrent MDD were excellent (*k*s = 0.88, 1.00, 1.00, *p*s < 0.001, respectively). Information about inter-rater reliability of other diagnoses from the MINI can be found in online SA3.

The pre-registered primary outcome was self-reported depression severity measured by the total score on QIDS-SR (Rush et al., 2003). Pre-registered secondary outcomes included interviewer-rated depression severity measured by total score on Hamilton Rating Scale for Depression-17 item version (HRSD-17; Hamilton, 1960), the anhedonic depression subscale of the Mood and Anxiety Symptom Questionnaire-Short Form (MASQ-SF; Watson et al., 1995), and the anxious arousal subscale of the MASQ-SF. A licensed psychologist provided weekly supervision for the HRSD-17 throughout the trial. Inter-rater reliability for the HRSD-17 was high [intraclass correlation coefficient (ICC) = 0.98]. Interviewers were blinded to participants' ABMT condition. The anhedonic depression and anxious arousal subscales of the MASQ-SF were included as secondary outcomes to examine ABMT effects on a wider range of symptoms than those measured by the QIDS-SR.

Target engagement was measured with an affective dot-probe assessment task with eye tracking using stimuli presented for 1000 ms. Participants were presented affective stimuli (happy and sad faces paired with neutral faces from the same actor) before identifying a probe (e.g. O or Q) using a button press. The assessment of attention bias target engagement was the number of trials where net fixation time was greater for sad than neutral stimuli. Stimuli used in the baseline and target engagement dot-probe assessments (Karolinska Directed Emotional Faces) were distinct from stimuli used during ABMT. Split-half internal consistency of affective dot-probe task at baseline was adequate [Spearman–Brown corrected *r* = 0.57, 95% confidence interval (CI) 0.43–0.68 for the number of trials where net fixation time was greater for negative than neutral stimuli; see online SA5 and Table SA1 for more details on the reliability of task outcomes]. This metric also had adequate 1 week stability (*r* = 0.54) in the assessment-only condition from baseline to week 1.

### Interventions

#### Attention bias modification training

Active ABMT presented pairs of stimuli to the right and left visual fields from two stimulus categories: sad or neutral facial expressions from the well-validated Pictures of Facial Affect (POFA) collection and dysphoric or neutral scenes from the well-validated International Affective Picture System (IAPS) collection (Ekman & Friesen, 1976; Lang, Bradley, & Cuthbert, 2005). Additional details on stimuli are provided in online SA4.

Each ABMT trial began with a central fixation cross for 1500 ms, followed by a pair of POFA or IAPS stimuli. ABMT POFA pairs were presented for 3000 ms, while IAPS pairs were presented for 4500 ms (due to the increased image complexity of IAPS images relative to POFA images and research showing that depression is associated with sustained attentional biases for dysphoric stimuli; Armstrong & Olatunji, 2012). Following image offset, either a single or double asterisk target appeared in the location of one of the two previously presented images and remained until a participant responded indicating the probe type or 10 s. In active ABMT, the target had an 80% probability of appearing in the location of the neutral stimulus. We selected 80% rather than a 100% probability in order for the task not to be highly transparent and to facilitate participant engagement with the task, consistent with our past study.

#### Sham ABMT

Consistent with prior research (e.g. Beevers et al., 2015; Li et al., 2016; Yang et al., 2015), sham and active ABMT were identical in all respects with one critical exception: after stimuli offset the target appeared with equal probability (50%) in the location of the neutral or the dysphoric stimulus. The *only* difference between sham and active ABMT was the probability with which the target appeared in the location of the neutral stimulus; thus, active ABMT putatively trains attention away from dysphoric information whereas sham ABMT has no such contingency. Other ABMT parameters were identical to our previous research (Beevers et al., 2015; Wells & Beevers, 2010).

#### ABMT dosage

Participants were scheduled to complete ABMT sessions twice a week in-clinic and three times per week at home for a total of 4 weeks. In-clinic training was longer than at-home training – nine blocks of 22 trials (198 trials total, approximately 25 min in length) compared to three blocks of 22 trials (66 trials total, approximately 8 min in length) for a total of 2224 trials across all training sessions. In-clinic training provided participants two breaks to reduce fatigue. At-home training was shorter in length to facilitate adherence and disperse training. The ABMT intervention was programed to only run if presented full screen to maximize the likelihood of engagement by the participant. Additional details regarding the training can be found in the study protocol (Hsu et al., 2018) and online SA6.

#### ABMT randomization

Participants were randomized using a randomized block design. Within each block, defined by the cross-product of depression severity (below or above a QIDS score of 20), gender (men, women, or non-binary), and antidepressant usage (taking or not taking), the sequence of treatment assignments was generated by repeatedly and randomly permuting the order of the three conditions, such that each condition was guaranteed near-equal representation within each block. Details regarding the implementation of the study blind can be found in online SA7.

### Procedure

Participants were initially screened online for potential eligibility, then completed a phone screening with trained study staff, and finally attended an in-person baseline assessment before enrollment and randomization. After participants provided written informed consent for the study, they completed the eye-tracking affective dot-probe task, questionnaires, and clinician-administered interviews. In a second session, 1–3 days later, participants completed several measures not included in this report (including an fMRI assessment) that were not primary or secondary outcomes. Participants were then randomized into one of the three conditions (i.e. active ABMT, sham ABMT, or assessments only). If participants were randomized into an ABMT condition, they immediately completed their initial training session with study staff present who demonstrated how to access the training, reviewed training procedures, and answered participant questions.

After these baseline measures, participants completed 4 weekly follow-up assessments. Participants were compensated after each visit, up to a total of $275 for all visits. All study procedures were approved by the University of Texas's Institutional Review Board. Throughout the study, a data safety monitoring board oversaw the study and was provided annual updates by the study team regarding the number and nature of adverse events (of which there were none).

### Data analysis

We used linear mixed-effects regression to test study hypotheses and assess target engagement. We modeled symptom severity and negative attention bias as a function of a fixed effect of time, measured as a continuous variable (in units of weeks) from the date of the final baseline assessment (which was immediately followed by the start of training for the active and sham ABMT groups), a fixed effect of training assignment conditioned on time (time × training interaction), a random slope of time, and a random intercept for participant (Hsu et al., 2018). Baseline measures obtained prior to randomization were modeled as a covariate and the unconditional effect of training was assumed to be 0 and not estimated, which specifies that groups should share a common intercept at the start of training (when time = 0).

For target engagement, a generalized linear mixed effects model was fit to the trial-level binomial outcome of whether or not gaze was directed primarily (>50%) toward or away from the sad face. Given the complexity of longitudinal mediation analyses, future research will conduct longitudinal mediation analyses to determine whether change in negative attention bias mediates an association between ABMT and depression symptom change.

Effect sizes were calculated using Equation (9) from Feingold (2013):
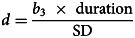
where *b*_3_ is the beta coefficient for the group × time interaction, duration is the number of weeks, and SD is the pooled within-group standard deviation for the observed outcomes at the final time point. Thus, this quantity uses the interaction term from the linear mixed effects regression to estimate the effect size of the predicted group difference after 4 weeks of training.

Importantly, we used a data blind analysis approach (MacCoun & Perlmutter, 2015): training condition assignments were permuted when testing model assumptions so that observed results would not influence decision making. When the model was finalized, the permuted training condition data were replaced with the actual data and models were analyzed. The analyses, which were decided *prior to unblinding*, represent a minor modification from our pre-registered data analysis plan. The rationale for this modification is explained in online SA8. Results from the pre-registered model are presented in online SA9, which leads to the same overall conclusions.

## Results

### Participant characteristics

The participants enrolled in the study were primarily female, non-Hispanic White, not on psychiatric medication, and in early adulthood. Table 1 presents the demographic characteristics for the sample at the baseline assessment. Gender was not associated with treatment response for the primary outcome (see online SA16).

**Table 1.** Sample characteristics at enrollment

	No training	Sham training	Active training
Total *N*	48	49	48
Mean age	26.1 (s.d. = 6.1)	25.3 (s.d. = 5.9)	24.4 (s.d. = 5.4)
Current MDD	38 (79%)	36 (73%)	34 (71%)
Past MDD	43 (90%)	36 (73%)	38 (79%)
On psychiatric med	11 (23%)	11 (22%)	12 (25%)
Symptom severity			
Mean QIDS	15.8 (s.d. = 2.4)	16.1 (s.d. = 2.8)	16.2 (s.d. = 2.2)
Mean HRSD	16.3 (s.d. = 5.5)	16.7 (s.d. = 5.1)	16.5 (s.d. = 5.7)
Mean MASQ anxious	9.5 (s.d. = 6.6)	9.9 (s.d. = 6.8)	9.8 (s.d. = 7)
Mean MASQ anhedonic	31.5 (s.d. = 6.6)	31.8 (s.d. = 5.9)	30.3 (s.d. = 7.2)
Ethnicity			
Hispanic or Latino	11 (23%)	14 (29%)	13 (27%)
Non-Hispanic	37 (77%)	35 (71%)	35 (73%)
Gender			
Male	10 (21%)	11 (22%)	11 (23%)
Female	36 (75%)	38 (78%)	37 (77%)
Other	2 (4%)	0 (0%)	0 (0%)
Race			
White	30 (62%)	29 (59%)	31 (65%)
American Indian/Alaska Native	1 (2%)	1 (2%)	0 (0%)
Asian	7 (15%)	8 (16%)	11 (23%)
Black or African American	4 (8%)	2 (4%)	1 (2%)
Multiracial	4 (8%)	5 (10%)	2 (4%)
Unknown or not reported	2 (4%)	4 (8%)	3 (6%)

### ABMT adherence

Median adherence was 74% [1646 trials, interquartile range (IQR) = 44–85%] for active ABMT and 74% (IQR = 47–91%) for sham training. There was a significant linear decline in training adherence by approximately 5% per week (*p* = 0.03). The rate of decline in adherence was significantly greater for at-home training, which saw an additional 9% decline per week as compared to clinic training (*p* < 0.001). Training groups did not significantly differ in overall adherence (*p* = 0.57) or change in adherence over time (*p* = 0.41). Additional information is provided in online SA10.

### Primary outcome – QIDS-SR

**Fig. 2.**
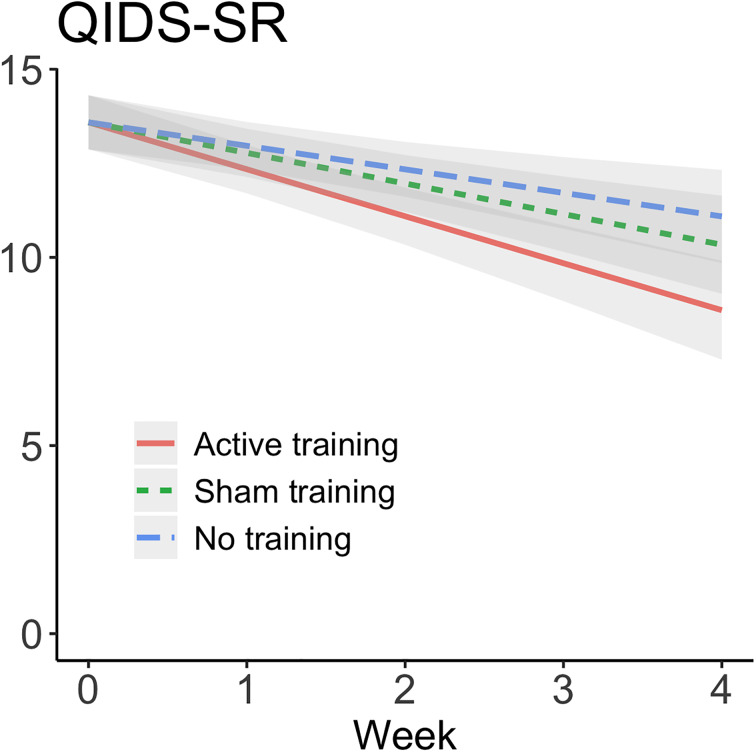
Change in self-reported depression symptoms (QIDS-SR) over time presented by training condition. Effects from linear mixed effects regression with 95% confidence bands. Reductions in self-reported depression symptom severity was greater for active ABMT than assessment-only (*d* = −0.57, *p* = 0.008) and sham ABMT (*d* = −0.41, *p* = 0.067). QIDS-SR, Quick Inventory Depression Scale-Self-Report version. Means, standard deviations, and *N*s at each time point are available for all primary and secondary outcomes in online Supplementary materials.

Regression model parameters (with the assessment-only condition as the reference group) are provided in Table 2. There was a significant group-by-time interaction, as active ABMT predicted an additional 0.62 (s.e. = 0.23, *p* = 0.008) points-per-week reduction in self-reported depression symptoms compared to the assessment-only group (*d* = −0.57). By comparison, sham ABMT only predicted an additional 0.19 (s.e. = 0.23, *p* = 0.41) points-per-week reduction compared to the assessment-only group, which was not a significant difference (*d* = −0.17). Comparing active to sham ABMT, active ABMT predicted an additional 0.44 (s.e. = 0.24, *p* = 0.067) points-per-week-reduction (*d* = −0.41; see online SA11 and Table SA2 for more details). The ABMT effects for self-reported depression are depicted in Fig. 2.

**Table 2.** Linear mixed effect modeling output

Outcome	Fixed effect estimate (s.e.)	*t* value	*p* value
*Primary outcome* – *QIDS-SR*			
Pre-treatment QIDS-SR	0.80 (0.12)	6.98	<0.001
Time	−0.63 (0.18)	−3.50	<0.001
Time × Active ABMT	−0.62 (0.23)	−2.72	0.008
Time × Sham ABMT	−0.19 (0.23)	−0.82	0.412
*Secondary outcome* – *HRSD-17*			
Pre-treatment HRSD-17	0.62 (0.06)	9.65	<0.001
Time	−0.92 (0.24)	−3.79	<0.001
Time × Active ABMT	−0.72 (0.31)	−2.28	0.025
Time × Sham ABMT	−0.02 (0.31)	−0.07	0.946
*Secondary outcome* – *MASQ-AD*^a^			
Pre-treatment MASQ-AD	0.86 (0.10)	9.02	<0.001
Time	0.08 (0.05)	1.67	0.097
Time × Active ABMT	−0.01 (0.07)	−0.18	0.858
Time × Sham ABMT	−0.09 (0.07)	−1.29	0.201
*Secondary outcome* – *MASQ-AA*^b^			
Pre-treatment MASQ-AA	0.10 (0.01)	10.21	<0.001
Time	−0.16 (0.05)	−3.60	<0.001
Time × Active ABMT	−0.02 (0.06)	−0.27	0.785
Time × Sham ABMT	0.05 (0.06)	0.76	0.448

*Note*: All group comparisons were relative to the assessment-only group.

a Beta coefficients reported are in reversed square-root units of difference due to skew.

b Beta coefficients reported are in square-root units of difference due to skew.

### Secondary outcomes

#### HRSD-17

**Fig. 3.**
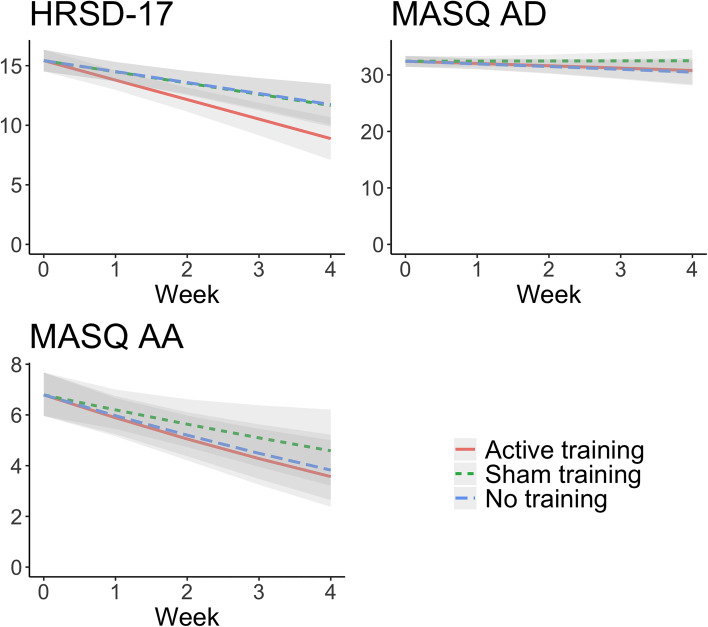
Change in the secondary outcomes (HRSD-17, MASQ-AD, MASQ-AA) over time presented by training condition. Effects from linear mixed effects regression with 95% confidence bands. Reductions in interviewer-rated depression symptom severity was greater for active ABMT than assessment-only (*d* = −0.49, *p* = 0.025) and sham ABMT (*d* = −0.42, *p* = 0.033). No training condition differences were observed for anhedonic or anxious arousal symptoms. HRSD-17, Hamilton Rating Scale for Depression-17 item; MASQ-AD, Mood and Anxiety Symptom Questionnaire-Anhedonic Symptoms; MASQ-AA, Mood and Anxiety Symptom Questionnaire-Anxious Arousal.

There was a significant group-by-time interaction, as active ABMT predicted an additional 0.72 (s.e. = 0.31, *p* = 0.025) points-per-week reduction in interviewer-rated depression symptoms compared to the assessment-only group (*d* = −0.49). By comparison, sham ABMT only predicted an additional 0.02 (s.e. = 0.31, *p* = 0.946) points-per-week reduction compared to the assessment-only group, which was not a significant difference (*d* = −0.01). Comparing active to sham ABMT, active ABMT predicted an additional 0.69 (s.e. = 0.32, *p* = 0.033) points-per-week reduction (*d* = −0.42). See Fig. 3 and Table 2 for full model results.

#### MASQ-SF

Anhedonic and anxious arousal symptoms, as measured by the MASQ-SF, did not change significantly over time, or in response to ABMT (see Fig. 3 and Table 2 for full model results). A table of ABMT effect sizes for all outcomes is presented in online Supplementary Table SA3 (means and s.d.s are presented in online Supplementary Table SA4).

### Target engagement

The time × group interaction indicated that active ABMT led to a reduction in the odds of a trial where gaze was greater for sad than neutral stimuli relative to assessment-only (OR = 0.9, *p* = 0.066) and sham ABMT (OR = 0.91, *p* = 0.075), although the pairwise comparison was not statistically significant. In contrast, the odds of eye gaze bias toward sad stimuli for sham ABMT remained equivalent to assessment-only (OR = 1, *p* = 0.97; see online SA14). Using a more traditional reaction time (RT) measure of bias, we did not find a significant time × group interaction (*p* > 0.056 for all pairwise comparisons; see online SA14).

### Post-hoc, follow-up analyses

#### Completer analysis

We examined post-training differences between completers in each group (*N* = 116), covarying for pre-treatment symptom severity. Individuals completing active ABMT showed a significant reduction (*d* = −0.63; *p* = 0.002) in QIDS-SR relative to the assessment-only group and a moderate reduction (*d* = −0.39; *p* = 0.054) relative to the sham ABMT group (for additional details, see online SA13).

#### Effects of training on remission rates between groups

Remission rates for the QIDS-SR (scoring 5 or less) ranged between 26% (95% CI 14–43) for the active ABMT group, 13% (95% CI 6–28) for the sham ABMT group, and 6% (95% CI 2–19) for the assessment-only group. Using logistic regression, we found that after controlling for pre-treatment severity, active ABMT predicted significantly greater odds of remission than the assessment-only group (OR = 5.09, 95% CI 1.36–25.0, *p* = 0.024) but not significantly greater than the sham ABMT group (OR = 2.29, 95% CI 0.72–7.82, *p* = 0.200).

#### Effects of training on treatment response rate between groups

Response rates for the QIDS-SR (at least 50% improvement from baseline) varied between 46% (95% CI 31–62) for the active ABMT group, 24% (95% CI 13–40) for the sham ABMT group, and 19% (95% CI 10–34) for the assessment-only group. Using logistic regression, active ABMT predicted significantly greater odds of response than both the assessment-only group (OR = 3.54, 95% CI 1.32–10.2, *p* = 0.014) and the sham ABMT group (OR = 2.76, 95% CI 1.03–7.76, *p* = 0.047).

## Conclusion

An intervention designed to direct attention away from dysphoric stimuli (i.e. active ABMT) was associated with significant reductions in both self-reported and interviewer-rated depression symptom severity during a 1-month acute treatment period compared to an assessments-only comparison condition (*d*s were 0.57 and 0.49, respectively). Active ABMT also reduced self-reported and interviewer-rated depression to a greater extent than sham ABMT (*d*s were 0.41 and 0.42, respectively), although the comparison for self-reported depression symptoms was not statistically significant (*p* = 0.067). These effect sizes are similar to prior research, although the most recently published meta-analysis indicates that ABMT effects can be inconsistent and at risk for bias (Fodor et al., 2020).

Given prior mixed evidence for the efficacy of ABMT for depression (e.g. Beevers et al., 2015; Yang et al., 2015), why might this trial have found positive results? Unlike previous ABMT studies for depression, this study recruited individuals who were putatively most likely to benefit from this type of intervention: adults with elevated depression *and* at least a moderate level of negative attention bias. Although speculative, this may be a key difference that explains why active ABMT was associated with a reduction in depression symptoms. Indeed, prior research in anxiety has shown that participants with a pre-treatment attention bias were more likely to respond to ABMT (Fox, Zougkou, Ashwin, & Cahill, 2015). However, selecting individuals with an initial attention bias requires psychometrically-validated tasks and metrics, a well-debated issue in this area of research (Parsons, Kruijt, & Fox, 2019). In addition, we used longer presentation times for stimuli in ABMT, which more closely matches free-viewing paradigms and the biases observed in those studies (Suslow et al., 2020). Consequently, the maintenance and disengagement biases observed in depressed individuals may have been better engaged with this training format; additional empirical research is needed to test this hypothesis.

Secondary outcomes suggest that our ABMT program may have relative specificity with regard to symptom change. Active ABMT decreased general depression symptom severity (both self-report and clinician-rated) but not the self-reported measures of anhedonia or anxious arousal. One possible explanation for the specificity of effects for ABMT is that the late-stage negative attentional bias targeted in this study may differ from the attentional biases that might maintain anhedonia or anxiety (Armstrong & Olatunji, 2012; Peckham et al., 2010). We also did not use threatening stimuli to target anxiety, unlike other variations of ABMT found to be efficacious for anxiety (Bar-Haim, 2010). Alternatively, many participants were not experiencing heightened anxious arousal from the outset of the study.

Individuals in the active ABMT group showed greater remission and response rates than individuals in the sham ABMT and assessment-only groups. The remission and response rates for individuals receiving 4 weeks of active ABMT are relatively comparable to longer courses of antidepressant medication (e.g. Rush et al., 2006) and cognitive behavioral therapy (e.g. Driessen et al., 2013; Hollon et al., 2014). Perhaps more importantly, ABMT targets a putative maintenance factor for depression (i.e. negative attentional bias) that, at present, has not shown to be well-targeted in other therapeutic interventions for depression (cf. Harmer et al. 2009; Wells, Clerkin, Ellis, & Beevers, 2014). ABMT may be a useful tool to implement in clinical settings given this potential unmet therapeutic need.

Our study had a number of strengths relative to previous studies of ABMT for depression (Fodor et al., 2020). The inclusion of an assessment-only condition controls for symptom reduction due to non-specific effects (e.g. enrollment in a clinical trial, interaction with study staff, or spontaneous remission). Only one prior study has tested active *v.* sham ABMT *v.* an assessment-only control (Yang et al., 2015). Furthermore, our study protocol was pre-registered, and we performed data-blind analyses; thus, modeling decisions were made prior to revealing the study results. Finally, our study had a relatively low risk of bias (Fodor et al., 2020), as we employed random sequence generation for allocation of participants to treatment groups, concealed treatment allocation by masking participants, personnel, and outcome assessors to training type assignment, included all enrolled and randomized participants into analyses, and reported on all prospectively registered primary and secondary outcomes.

Important limitations include the recruitment of few participants with severe levels of depression and suicidality, a focus on acute response to ABMT (the long-term effects of ABMT remain unknown, including to what extent ABMT confers protective effects against future episodes of depression), a narrow age range, and a higher than anticipated rate of attrition. Although our sample is more diverse than previous studies of ABMT in depression, it is still a predominantly WEIRD (white, educated, industrialized, rich, democratic) sample (Henrich, Heine, & Norenzayan, 2010) and the stimuli used for the dot-probe assessment solely consisted of White faces (although recent research suggests that racial groups do not attend to face stimuli differently if the face stimuli vary by race; Gonzalez & Schnyer, 2018). Finally, as we selected our sample based on pre-treatment bias and self-reported depression severity, overall regression to the mean may have also made it more difficult to detect group-related treatment effects.

Finally, the effect of ABMT on target engagement was weaker than symptom change, possibly due to the measurement challenges associated with attention bias (Parsons et al., 2019). This last point underscores the continued threat reliability issues pose to scientific progress in clinical science. The study of attentional biases and ABMT would benefit from identifying more reliable outcomes, novel *and* psychometrically validated tasks, and the use of multiple measures of attention bias to enable latent variable modeling of bias as potential ways forward. In addition, alternative approaches to modifying negative attentional bias (e.g. Godara, Sanchez-Lopez, & De Raedt, 2019; Shamai-Leshem, Lazarov, Pine, & Bar-Haim, 2020) may be worth exploring as stronger empirical tests of whether modifying negative attentional bias reduces depression symptom severity over time if these methods can achieve greater target engagement than what was demonstrated in this study.

Aside from addressing the limitations outlined above, a number of future directions exist for ABMT for depression. Identifying behavioral and neurobiological mediators of treatment response can further clarify the relevant processes maintaining depression that are modified in ABMT (Beevers et al., 2015; Hilland et al., 2020). In addition, other studies suggest that attention bias modification may have a differential impact on symptoms of depression (Kraft et al., 2019). Finally, our findings suggest exploring the possibility that ABMT for depression might be a low-effort, scalable intervention that can be added as an adjunctive intervention for real-world treatment settings.

In conclusion, findings are consistent with cognitive models of depression that posit negative attention bias as a maintenance factor for the disorder (Disner et al., 2011; Joormann & Vanderlind, 2014). Training effects for active ABMT were specific to broad measures of depression, as it did not significantly alter the specific symptom dimensions of anhedonia or anxious arousal. This study suggests that negative attention bias may have an important role in the maintenance of depression and that ABMT has the potential to be a scalable intervention for depressed individuals with a negative attention bias.

## Supplementary material

The supplementary material for this article can be found at https://doi.org/10.1017/S0033291721000702.
