# Supplementary material for: Efficacy of attention bias modification training for depressed adults: a randomized clinical trial
Source: Psychol Med. 2021 Mar 26;52(16):3865–73. Online ahead of print. doi: 10.1017/S0033291721000702 (PMC8464627; doi:10.1017/S0033291721000702)
Supplement: Supplementary file 1 [file S0033291721000702sup001.pdf]

Supplemental Appendix

Table of Contents

1. Analysis Scripts, Analysis Reports, and Data Sharing

2. Reliability for the QIDS-SR

3. Inter-rater reliability for the MINI clinical interview

4. Dot-Probe Stimulus Presentation

5. Split-half reliability of attention bias assessment outcomes

6. Additional in-lab attention bias modification training details

7. Implementation of the Study Blind

8. Data analytic approach

9. Results of original, pre-registered data analytic plan

10. Training Compliance

11. Results of linear mixed effects modeling for the active versus sham ABMT comparison
  Table SA2. Linear Mixed Effect Modeling Output (with treatment contrasts against sham ABMT).

12. Table of Effect Sizes for ABMT
  Table SA3. Effect size (d) for each ABM comparison for the primary and secondary outcomes.
  Table SA4. Outcome Means, (SDs), Ns at each assessment.

13. Sensitivity Analyses: ANCOVA of post-training differences among completers, covarying for pre-training severity

14. Target Engagement Analyses

15. Treatment effects covarying for adherence

16. Exploring the relation between gender and treatment response

1. Analysis Scripts, Analysis Reports, and Data Sharing

To facilitate transparency, results of all analyses reported in this manuscript can be found in the Dataverse for the Mood Disorders Laboratory at the University of Texas at Austin. Here are the locations of the following elements:

Project dataverse: https://dataverse.tdl.org/dataverse/r33-attntrain

Analysis reports: https://doi.org/10.18738/T8/UWKEFM

Data: https://nda.nih.gov/study.html?id=660

The analysis reports are RMarkdown documents that contain the R code with its associated results that were used to generate the results presented in this manuscript. The reports also contain additional follow-up analyses (e.g., sensitivity analyses) that were performed but not reported due to space limitations. All primary results presented in the manuscript can be cross-checked with the results presented in those reports. The supplemental materials (below) also contain key findings from these analysis reports so that the interested reader does not have to search through the files in the Dataverse.

All analyses reported in the article and supplemental materials were implemented in R (version 4.0).[1] Our code made extensive use of the *tidyverse*[2] packages *dplyr*, *purrr,* and *tidyr* for general data extraction and transformation. The *itrak* package was used to process eye-tracking data and compute attention bias metrics. The *lme4*[3] package was used to fit linear mixed effects regression models, and the *lmerTest*[4] package was used to calculate inferential statistics. The *DHARMa*[5] package was used to plot residuals and test assumptions from the linear mixed effects models. Figures were generated using the packages *effects*[6], *ggplot2*[7] and *patchwork*.[8]

2. Reliability for the QIDS-SR

The QIDS-SR cronbach alphas ranged from .23 to .78 across assessments. The QIDS poor internal consistency at baseline is likely an artifact of a truncated distribution associated with the inclusion criteria.

3. Inter-rater reliability for the MINI clinical interview

Inter-rater agreement (Fleiss' Kappa) for current MDD, lifetime MDD, and recurrent MDD were excellent (*k*s = 0.88, 1.00, 1.00, *p*s < .001, respectively). Agreement for Generalized Anxiety Disorder, Lifetime Panic Disorder, and Current Panic Disorder were moderate to high (*k*s = 1.00, 1.00, and 1.00, *p*s < .001). Agreement for several diagnoses (e.g., Agoraphobia, Anorexia, Bipolar Disorder, Bulimia, Obsessive-Compulsive Disorder, Social Anxiety Disorder, Substance Use Disorder, Post-Traumatic Stress Disorder, Mood Disorders with Psychotic Features) was not computed because participants with these disorders were not present in the reliability subset, which prevented examination of agreement. These disorders were not common in the full sample, likely due in part to low prevalence rates in general and study exclusion criteria.

4. Dot-Probe Stimulus Presentation

4.1. Affective dot-probe assessment. Stimuli consisted of images of happy (12), sad (12), and neutral (24) facial expressions from the Karolinska Directed Emotional Faces database (KDEF[9]). Each emotional face was paired with the neutral expression of the same actor. Stimuli (subtending 2° by 4° visual angle) were presented to the left and right sides of the visual field against a grey background (RGB: 110,110,110), with a center-to-center distance of 480 pixels (5° by 4° visual angle). The location and presentation of the stimulus pair varied randomly throughout the task. Stimulus-pairs were randomly presented four times within each block to counterbalance their location. To minimize variation of luminance throughout the task, stimuli background were removed and replaced to match background color. Stimuli did not vary in terms of luminance (12.0 cd/m^2).

4.2. Capture of gaze behavior. Gaze behavior was recorded at a rate of 500 Hz using a video-based eye tracker (Eye-Link 1000 Plus Desktop Mount; SR Research, Osgoode, Ontario, Canda). Before the presentation of practice trials, participants completed a 13-point calibration routine in order to map eye position to screen coordinates. The calibration process was then validated with a 13-point retest routine. The calibration process was accepted if there was less than a 0.5 degree visual angle between initial calibration and subsequent validation. After the initial calibration process, quality of eye tracking was maintained through drift checks between each trial. Participants were required to maintain their gaze on the fixation cross before the next set of stimuli was presented; if fixation was not detected, a single-point drift correction procedure was initiated. All stimuli were normalized to mean luminance (12.0 cd/m^2).

4.3. Attention bias training. Pictures of Facial Affect (POFA) stimuli consisted of neutral (12) and sad (12) faces. Each sad POFA face was paired with the neutral expression of the same actor. International Affective Picture System (IAPS) stimuli consisted of dysphoric (10) and neutral (10) images. Stimuli-pairs (IAPS: 19° by 14° visual angle, POFA: 13° by 19° visual angle) were presented to the left and right sides of the visual field against a grey background (RGB: 110,110,110), with a center-to-center distance of 800 px (27° by 20° visual angle). Location and presentation of the IAPS and POFA stimulus pair varied randomly throughout the task. Stimuli-pairs did not vary in terms of luminance (12.0 cd/m2).

5. Split-half reliability of attention bias assessment outcomes

Bootstrapped split-half reliability for attention bias assessment outcomes are provided below in Table SA1.

Table SA1. Bootstrapped split-half reliability of attention bias assessment outcomes

**Outcome**	***r*, uncorrected (95% CI)**	***r*, SB corrected (95% CI)**	**rho, uncorrected (95% CI)**	**rho, corrected (95% CI)**
Mean bias, happy (RT)	.71 (.64 .78)	.83 (.78 .87)	.55 (.46 .64)	.71 (.63 .78)
Mean bias, happy (ET)	.50 (.38 .60)	.66 (.55 .75)	.33 (.22 .44)	.50 (.36 .61)
Percent toward, happy (RT)	.55 (.47 .63)	.71 (.64 .77)	.50 (.41 .58)	.66 (.58 .74)
Percent toward, happy (ET)	.45 (.36 .54)	.62 (.53 .70)	.32 (.21 .42)	.48 (.35 .59)
Variability, happy (RT)	.87 (.84 .90)	.93 (.91 .95)	.85 (.82 .89)	.92 (.90 .94)
Variability, happy (ET)	.91 (.89 .93)	.95 (.94 .96)	.88 (.85 .91)	.93 (.92 .95)
Mean bias, sad (RT)	.73 (.65 .79)	.84 (.79 .88)	.60 (.51 .68)	.75 (.68 .81)
Mean bias, sad (ET)	.40 (28 .52)	.57 (.43 .68)	.15 (.04 .27)	.27 (.08 .43)
Percent toward, sad (RT)	.58 (.50 .65)	.73 (.67 .79)	.51 (.43 .60)	.68 (.60 .75)
Percent toward, sad (ET)	.26 (.16 .37)	.42 (.27 .54)	.10 (.00 .22)	.19 (-.01 .36)
Variability, sad (RT)	87 (.83 .90)	.93 (.90 .95)	.87 (.84 .90)	.93 (.91 .95)
Variability, sad (ET)	.92 (.89 .94)	.96 (.94 .97)	.89 (.85 .91)	.94 (.92 .95)

6. Additional in-lab attention bias modification training details

The in-lab training consisted of 198 trials (22 trials x 9 blocks) lasting approximately 20 min. Participants were seated in an illuminated room (12.0 cd/m2), 45 cm from the computer screen. Before the task, participants' position and distance from the screen were assessed by an in-house automated procedure using a webcam connected to the training computer. This information was presented to the participant as a visual parallax. The participant would progress only if the participant was aligned with the webcam and their face was detected.

If both position and distance were not maintained for a duration window of 10 sec, this initial calibration attempt was considered a failure. After two failed attempts or a total duration of 60 sec, a behavioral version of the same task was instead employed. The parallax was followed by a nine-point calibration routine used to map eye position to screen coordinates. After completing calibration, participants were informed that the task would soon begin and all instructions would be presented on the monitor.

Participants were instructed to view the images naturally. Further, they were also instructed to look at the fixation cross prior to each trial in order to standardize the starting location of their gaze. The task began with a series of 20 practice trials, using IAPS and POFA images not included with test stimuli. Each trial began with the appearance of a central fixation cross (FC). Participants were required to maintain gaze of central fixation (subtending 1° by 1° visual angle) for 1500 msec. Immediately following fixation, a stimulus pair appeared for either 3 (POFA) or 4.5 (IAPS) sec. Location of stimuli (left or right side of visual field) was randomized with equal frequency. Following stimulus offset, a probe appeared (single or double-asterisk) in place of one of the stimuli. Participants were asked to indicate the type of probe by pressing "8" if one asterisk and "9" if two asterisks.

After their response, the probe disappeared before beginning the next trial. IAPS and POFA stimuli appeared a total of 12 and 10 times across the block, respectively. The 3rd and 6th block were followed by a self-paced break.

7. Implementation of the Study Blind

The randomization sequences generated for stratified blocks in this study were stored in a private, backend data structure connected to a public frontend Shiny dashboard through which researchers entered the participant's ID and clicked an "Enroll" button, which resulted in a unique random 3-character string (e.g., J89) being written to the participant's record in REDCap, without ever displaying the actual treatment assignment or even a dummy variable (e.g., A, B, C) for that assignment. Thus, even if a researcher somehow gained awareness of one participant's assignment, this would not unblind anyone else, nor could a researcher consciously or unconsciously form a hypothesis about, for example, what condition the treatment assignment variable refers to. The computer program administering the training would automatically retrieve this unique string and match it to a private backend lookup table to deliver the participant's training (sham or active), and personnel responsible for scheduling were provided a partial lookup table that indicated only which assignment codes should receive training vs. assessments only. The full lookup table was only available to the study's statistician and programmer, and the blind was not broken until all data were collected and a data-blind analysis had been completed.

8. Data analytic approach

Model specification. In specifying the random effects of the models, we followed the "keep it maximal" approach advocated by Barr and colleagues by first attempting to fit a model with a random intercept for each participant, a random slope over time for each participant, and the correlation between the two.[10] In the event that this model had issues with singularity or non-

convergence, we had planned to simplify the random effects specification by assuming independence (no correlation) between intercept and slope and, if this also resulted in a problematic fit, to omit the random slope, leaving only the random intercept. Simplification proved unnecessary for the models presented in the manuscript; all of the effects presented in Table 2 were derived from models that included a correlated random slope and random intercept. T-tests of model coefficients were performed using Satterthwaite's method as implemented in the 'lmerTest' package.[4] In addition, as a sensitivity analysis, we report below the results of a simple ANCOVA predicting training differences at the final time point only, covarying for pre-training baseline differences. This is a more traditional outcome than rate of change but is potentially biased because it excludes participants who dropped out of the study before the 4-week time point, whereas the rate-of-change analysis makes use of all available followup data.

Linear model assumptions. After fitting a regression model for each outcome, we examined linear model assumptions (e.g., normally distributed outcomes, homoscedasticity, normally distributed residual errors). Models for two of the outcomes, the MASQ anhedonic depression and anxious arousal subscales, significantly violated these assumptions owing to substantial negative and positive skew of these respective outcomes. A square-root transform of anxious arousal and reversal of the anhedonic depression scale followed by square-root transform remedied these problems (Bartlett, 1947).

8.1. Pre-registered analytic approach and rationale for deviation. The *a priori* model specification from our pre-registered protocol was to model the severity of depression symptoms as a function of the fixed effect of time, measured as a continuous variable (in units of weeks) from the start of training (or, equivalently, the day of baseline fMRI for the assessment-only group), an unconditional fixed effect of training assignment, and the fixed

effect of training assignment conditioned on time (the time × training interaction).[11] In this design, often referred to as a longitudinal data analysis (LDA), pre-treatment measurements are modeled as part of the outcome variable alongside post-treatment measures.[12] One obvious problem with this approach is that, logically speaking, a baseline measurement cannot be a response to treatment, but the above model specification permits precisely that, attributing variance in *pre-training* symptom severity to *future* training assignment.[13] This is also equivalent to testing for a significant baseline difference, a practice that has been rightly discouraged in the CONSORT statement on the grounds that it is unnecessary in the context of a randomized trial and potentially misleading.[14] Any group differences at baseline *must* be due to chance, so it is illogical to allow a model to predict anything other than group equivalence at Time 0 of a randomized clinical trial. More logically defensible modeling options include the ANCOVA approach, in which baseline values are used as a covariate to predict subsequent measures, and the constrained longitudinal data analysis (cLDA) model, in which baseline values are considered part of the response outcome but baseline means are constrained to be equal across treatment groups.[15]

The differences between these approaches is largely philosophical since they tend to yield similar conclusions and even identical effects estimates under certain conditions (e.g., ANCOVA and cLDA are equivalent when there are no missing data). However, the question of which approach to use is not merely academic; Coffman et al. show an example in which p-values could range from 0.006 to 0.15, depending on the analysis method used, and they review evidence that both the ANCOVA and cLDA approaches are superior to the unconstrained LDA model that we preregistered.[12] Therefore, we thought it prudent to critically evaluate these

modeling approaches and specify prior to data unblinding which one should take precedence if there was non-consensus regarding whether the null hypothesis should be rejected.

We did this in the context of a data-blind analysis, in which we fit models using simulated group assignments (see 1.0-jds-blind-data-plan.html in the associated dataverse: https://doi.org/10.18738/T8/UWKEFM). This exercise made it clear that both the pre-registered LDA and the purportedly superior cLDA approaches, which both treat baseline as part of the response vector, violated an assumption that baseline and post-baseline values are jointly multivariate normally distributed.[15] This happened because the QIDS-SR was used as an inclusion/exclusion criterion, which resulted in a baseline distribution that was skewed and truncated at the eligibility cutoff with far less variance than the subsequent measurements.

On the other hand, the standard ANCOVA approach — in which experimental groups are allowed to have different intercepts (i.e., a "main" effect of training) as well as different slopes (i.e., an interaction effect of training by time) and baseline is used as a covariate — loses information about the rate of change between start of training and the first post-baseline measurement at 1 week. Relatedly, information about group equivalence at the start of training is also lost, and our blind data analysis demonstrated that the standard ANCOVA failed to model the group mean trendlines as originating from a point of equivalence, even though our simulated group assignments had been chosen to ensure baseline equivalence. Given that we predefined our primary outcome as the difference in rate of change from baseline (as opposed to a treatment difference at the final time point), the inability of this approach to accurately estimate group slopes starting from baseline was unacceptable.

Our solution was to apply the constraining logic of the cLDA — equivalence of groups prior to start of training is built into the model as a given — but treat baseline values as a

covariate rather than as part of the dependent variable. Given that we determined this model specification to be logical and statistically sound, the results presented in the main body of the text correspond to our preferred approach. We also include the results from the preregistered approach below. In this case, both approaches yielded very similar conclusions regarding group differences in the rate of change.

9. Results of original, pre-registered data analytic plan

9.1 Primary outcome - QIDS-SR. There was a significant group by time interaction, as active ABMT predicted an additional 0.65 (SE = 0.24, p = .008) points-per-week reduction in self-reported depression symptoms compared to the assessment only group (d = -0.59). By comparison, sham ABMT only predicted an additional 0.27 (SE = 0.24, p = .272) points-per-week reduction compared to the assessment-only group, which was not a significant difference (d = -0.25). Compared to sham ABMT, active ABMT predicted an additional 0.38 (SE = 0.25, p = .125) points-per-week-reduction (d = -0.36).

9.2 Secondary outcome - HRSD-17. There was a significant group by time interaction, as active ABMT predicted an additional 0.70 (SE = 0.32, p = .029) points-per-week reduction in self-reported depression symptoms compared to the assessment only group (d = -0.48). By comparison, sham ABMT only predicted an additional 0.08 (SE = 0.32, p = .800) points-per-week reduction compared to the assessment-only group, which was not a significant difference (d = -0.05). Compared to sham ABMT, active ABMT predicted an additional 0.62 (SE = 0.32, p = .058) points-per-week-reduction (d = -0.38).

9.3 Secondary outcome - MASQ-SF. There was not a significant time × training group interaction in the models predicting the anhedonic depression or anxious arousal subscales of the MASQ-SF. Full model results can be found in 1.13-jds-secondary-analysis-

masq.html in the associated dataverse.

10. Training Compliance

10.1 Testing for group differences. Participants who received active training completed a median of 1650 trials (IQR = 990-1913), and participants who received sham training also completed a median of 1650 trials (IQR = 1056-2046). A Mann-Whitney U test showed no significant difference in the amount of training that each group received (U = 1099, p = 0.58). Furthermore, the 95% confidence interval for the difference in the amount of training between groups was [-264, 132]. Relative to the sample-wide median of 1650 trials, the bounds of this CI are equivalent to an 8-16% difference. Because we would regard less than a 20% difference as equivalent training, this 95% CI falls within the expected margin of equivalence [-330, 330].

10.2 Adherence to target training level. Participants were scheduled for a total of 8 in-clinic trainings. The very first training, which occurred at enrollment following the fMRI scan, was abbreviated to 66 trials, but otherwise in-clinic trainings were 198 trials in length. Thus, this amounts to an in-clinic training target of 66 + (7 * 198) = 1452 trials. Participants were also asked to complete a total of 12 at-home trainings, which amounts to a target of 12 * 66 = 792 trials. Therefore, percent adherence was calculated for each person as a fraction of 1452 + 792 = 2244 trials. The median participant was 74% (IQR = 44-85%) adherent to active training and 74% (IQR = 47-91%) adherent to sham training protocols.

11. Results of linear mixed effects modeling for the active versus sham ABMT comparison

Table SA1 presents the results for the linear mixed effects modeling using sham ABMT as the comparison condition. These models are identical to the models presented in Table 2 with the exception that the treatment contrasts specify sham ABMT as the reference level instead of assessment only. We prioritized the active ABMT vs assessment-only

comparisons in the main document because those are the contrasts that we were powered to detect and that we indicated would be our primary outcome in our pre-registration. We presented the results of the Time × Active ABMT contrasts from this table in the text of the manuscript as the basis for comparing active to sham ABMT. Full analysis scripts and results can be found in this project's dataverse: https://doi.org/10.18738/T8/UWKEFM

Table SA2. Linear Mixed Effect Modeling Output (with treatment contrasts against sham ABMT).

Outcome	Fixed Effect Estimate (Standard Error)	*t*-value	*p*-value
Primary Outcome - QIDS-SR			
Pre-treatment QIDS-SR	0.80 (0.12)	6.98	< .001
Time	-0.81 (0.19)	-4.36	< .001
Time × Assessment only	0.19 (0.23)	0.82	.412
Time × Active ABMT	-0.44 (0.24)	-1.85	.067
Secondary Outcome – HRSD-17			
Pre-treatment HRSD-17	0.62 (0.06)	9.65	< .001
Time	-0.94 (0.25)	-3.72	< .001
Time × Assessment only	0.02 (0.31)	0.07	.946
Time × Active ABMT	-0.69 (0.32)	-2.16	.033
Secondary Outcome – MASQ-AD[a]			
Pre-treatment MASQ-AD	0.86 (0.10)	9.02	< .001
Time	-0.00 (0.05)	-0.07	.949
Time × Assessment only	0.09 (0.07)	1.29	.201
Time × Active ABMT	0.07 (0.07)	1.08	.283

Secondary Outcome – MASQ-AA[b]			
Pre-treatment MASQ-AA	0.10 (0.01)	10.21	< .001
Time	-0.12 (0.05)	-2.43	.017
Time × Assessment only	-0.05 (0.06)	-0.76	.448
Time × Active ABMT	-0.06 (0.06)	-1.01	.316

Note: All group comparisons were relative to the sham ABMT group.
[a]beta coefficients reported are in reversed square-root units of difference due to skew
[b]beta coefficients reported are in square-root units of difference due to skew

12. Table of Effect Sizes for ABMT

This table contains effects sizes for all ABMT condition comparisons for the primary and secondary outcomes.

Table SA3. Effect size (*d*) for each ABM comparison for the primary and secondary outcomes.

Condition	QIDS-SR	HRSD-17	MSAQ-AD	MASQ-AA
Active ABMT vs Assessment Only	-0.57	-0.49	0.04	-0.05
Active ABMT vs Sham ABMT	-0.41	-0.42	-0.22	-0.24
Sham ABMT vs Assessment Only	-0.17	-0.01	0.25	0.19

Table SA4. Outcome Means, (SDs), Ns at each assessment.

Active Training	Sham Training	No Training
**QIDS**		

0	16.2 (2.2), N = 48	16.1 (2.8), N = 49	15.8 (2.4), N = 48
1	13 (3.8), N = 40	12.5 (4.1), N = 42	13.4 (3.8), N = 47
2	11.1 (4.3), N = 38	12.1 (4.7), N = 39	13.2 (5), N = 42
3	10 (4.3), N = 35	10.6 (4.6), N = 35	11.4 (5), N = 39
4	9 (4.3), N = 37	10.4 (4.2), N = 38	11.7 (4.4), N = 41

HRSD

0	16.5 (5.7), N = 48	16.7 (5.1), N = 49	16.3 (5.5), N = 48
1	14.2 (5.4), N = 41	15.6 (5.7), N = 41	14.2 (5.6), N = 47
2	12.9 (6.6), N = 38	13.1 (6.3), N = 37	14.2 (6.7), N = 42
3	10.2 (5.7), N = 33	12.9 (6.2), N = 35	12.2 (6.1), N = 40
4	9.3 (5.9), N = 38	12.1 (6.5), N = 38	11.8 (5.5), N = 40

MASQ Anxious Arousal

0	9.8 (7), N = 47	9.9 (6.8), N = 46	9.5 (6.6), N = 46
1	6.7 (4.8), N = 40	7.9 (6), N = 41	8 (6.3), N = 46
2	5.6 (5.4), N = 38	7 (4.9), N = 39	6.7 (5.5), N = 41
3	6.3 (6.2), N = 35	6.1 (4.9), N = 35	6.3 (6), N = 38

4	4.3 (4.7), N = 36	6.8 (5.2), N = 37	5.6 (5.5), N = 41

MASQ Anhedonic

0	30.3 (7.2), N = 47	31.8 (5.9), N = 46	31.5 (6.6), N = 46
1	30 (7.4), N = 40	31.8 (5.6), N = 41	32 (5.8), N = 46
2	29.2 (7.3), N = 38	31.1 (6.1), N = 39	30.5 (7.6), N = 41
3	28.5 (7), N = 35	30.3 (7.1), N = 35	30.3 (7), N = 38
4	28.7 (8.3), N = 36	31.2 (6.3), N = 38	29.1 (8.7), N = 41

13. Sensitivity Analyses: ANCOVA of post-training differences among completers, covarying for pre-training severity

This analysis used an ANCOVA of observed final QIDS-SR (no imputation of unobserved final outcomes) while covarying for baseline QIDS. For inclusion in this analysis, participants only needed to attend their final assessment week (N = 116), excluding the one participant who received their final assessment at nearly 8 weeks instead of the planned 4 weeks. Full model details are available in 1.11-jds-mood-primary-analysis.html, 1.12-jds-mood-secondary-analysis-hrsd.html, and 1.13-jds-mood-secondary-analysis-masq.html (https://doi.org/10.18738/T8/UWKEFM).

13.1 Primary outcome: QIDS-SR. There was a significant training condition effect, as active ABMT predicted that self-reported depression symptoms at post-training were 2.71 (SE = 0.85, p = .002) points lower than in the assessment only group (d = -0.63). By comparison, sham ABMT predicted a score that was 1.03 (SE = 0.84, p = .224) points lower than the assessment-

only group (*d* = -0.24). Compared to sham ABMT, active ABMT predicted a 1.68 (SE = 0.86, *p* = .054) point lower post-treatment score (*d* = -0.39).

13.2 Secondary outcome: HRSD-17. There was a significant training condition effect, with active ABMT predicted to produce a score that was 2.67 (SE = 1.23, *p* = .033) points lower than the assessment only group (*d* = -0.47). By comparison, sham ABMT was predicted to have a 0.14 (SE = 1.23, *p* = .91) points higher score than the assessment-only group (*d* = 0.02). Active ABMT was predicted to have a 2.81 (SE = 1.25, *p* = .026) point lower post-training score than sham ABMT (*d* = -0.45).

13.3 Secondary outcome: MASQ-AD. There was not a significant training condition effect. There was a predicted active ABMT - assessment only difference of -0.05 (SE = 0.28, *p* = .852) points in square-root units (*d* = 0.04). By comparison, sham ABMT was 0.41 (SE = 0.28, *p* = .144) square-root points *greater* than the assessment-only group (*d* = 0.30). Compared to sham ABMT, active ABMT was 0.36 (SE = 0.28, *p* = .214) square-root points lower (*d* = -0.26).

13.4 Secondary outcome: MASQ-AA. There was not a significant training condition effect, as active ABMT predicted a lower post-training score, -0.25 (SE = 0.23, *p* = .265) square-root units, than the assessment only group (*d* = -0.22). By comparison, sham ABMT predicted a 0.29 (SE = 0.23, *p* = .206) square-root points greater score than the assessment-only group (*d* = 0.28). Compared to sham ABMT, active ABMT predicted a -0.54 (SE = 0.24, *p* = .022) square-root point difference (*d* = -0.51).

14. Target Engagement Analyses

14.1 Definition of target engagement. As noted in our methods, a generalized linear mixed effects model examined whether ABMT, time, and their interaction was associated with change in a trial-level binomial variable of whether or not gaze was directed primarily (> 50%)

toward or away from sad stimuli. During peer review of this manuscript it was suggested that we consider using the original criterion (37.5% of trials with a negative bias) as a test for target engagement. To follow this suggestion, this would mean creating a binomial outcome of whether or not participants would still meet the eligibility criterion. In the end, we decided against this idea because the 37.5% criterion is not an inclusion criterion for high bias; it is an exclusion criterion for low bias to make sure that there is room for bias to improve. It is not meant to indicate the cutoff for a healthy vs. unhealthy amount of bias. Thus, we examined change in the odds of gaze being directed primarily toward sad stimuli as our primary measure of target engagement.

14.2 Exploratory target engagement analyses. The project's data repository (https://doi.org/10.18738/T8/UWKEFM) contains detailed reports of our target engagement analyses. Specifically, 1.02-jds-gaze-bias-blind-analysis-plan.html contains our rationale for how we selected our primary target engagement outcome using data blind analyses. Document 1.21-jds-bias-primary-analysis.html describes how we implemented our target engagement analyses. We then conducted exploratory analyses using other eye gaze and behavioral metrics of attention bias (documented in 1.22-jds-bias-secondary-analysis-RT.html and 1.23-jds-bias-secondary-analysis-itrak.html). Notably, most traditional metrics of attention bias did not significantly change with ABMT; however, a trial-level metric, attention bias variability, appears to change with active ABMT compared to the assessment only condition. This was an exploratory analysis so it is not included in the main outcome paper.

15. Treatment effects covarying for adherence

To further understand the relationship between training treatment outcome, we conducted post-hoc analyses with individuals that completed either active or sham ABMT that examined

how training group and adherence rate were associated with post-treatment symptom severity, also controlling for baseline symptom severity. Greater adherence predicted greater symptom reduction: for example, a 10% increase in adherence was associated with an additional 0.5 (SE = 0.2, p = .035) point decrease in QIDS score. After controlling for adherence, the active group was associated with a significantly greater decrease in QIDS score than the sham group (b = 1.74, SE = 0.87, p = .049).

16. Exploring the relation between gender and treatment response

Although underpowered, we conducted exploratory analyses to further examine the association between gender and treatment response to ABMT. We observed no significant gender x group x week or gender x week interactions, nor a main effect of gender in our primary outcome analyses. We followed with a subgroup analysis with women only; treatment effects appeared larger, as active ABMT condition significantly differed from the sham ABMT (p = .032, d = -0.58) and assessment only (p = .007, d = -0.70) conditions. In the original analyses with the full sample, the active ABMT condition outperformed the assessment only condition (p = .008, d = -0.57) but not the sham ABMT condition (p = .067, d = -0.41).

References

1 R Core Team. R: A Language and Environment for Statistical Computing. Vienna, Austria: R Foundation for Statistical Computing, 2020 https://www.R-project.org/.

2 Wickham H, Averick M, Bryan J, *et al.* Welcome to the tidyverse. Journal of Open Source Software. 2019; **4**: 1686.

3 Bates D, Mächler M, Bolker B, Walker S. Fitting Linear Mixed-Effects Models Using lme4. *J Stat Softw* 2015; **67**: 1–48.

4 Kuznetsova A, Brockhoff PB, Christensen RHB. lmerTest package: tests in linear mixed effects models. *J Stat Softw* 2017; **82**: 1–26.

5 Hartig F. DHARMa: Residual Diagnostics for Hierarchical (Multi-Level / Mixed) Regression Models. 2020 https://CRAN.R-project.org/package=DHARMa.

6 Fox J, Weisberg S. An R Companion to Applied Regression, 3rd edn. Thousand Oaks CA: Sage, 2019 http://tinyurl.com/carbook.

7 Wickham H. ggplot2: Elegant Graphics for Data Analysis. Springer-Verlag New York, 2016 https://ggplot2.tidyverse.org.

8 Pedersen TL. patchwork: The Composer of Plots. 2020 https://CRAN.R-project.org/package=patchwork.

9 Lundqvist D, Flykt A, Öhman A. Karolinska Directed Emotional Faces. 2015 DOI:10.1037/t27732-000.

10 Barr DJ, Levy R, Scheepers C, Tily HJ. Random effects structure for confirmatory hypothesis testing: Keep it maximal. *J Mem Lang* 2013; **68**: 255–78.

11 Hsu KJ, Caffey KD, Pisner D, *et al.* Attentional bias modification treatment for depression: Study protocol for a randomized controlled trial. *Contemporary Clinical Trials* 2018; **75**: 59–66.

12 Coffman CJ, Edelman D, Woolson RF. To condition or not condition? Analysing ‘change’ in longitudinal randomised controlled trials. *BMJ Open* 2016; **6**: e013096.

13 Senn S. Change from baseline and analysis of covariance revisited. *Stat Med* 2006; **25**: 4334–44.

14 Moher D, Hopewell S, Schulz KF, *et al.* CONSORT 2010 Explanation and Elaboration: Updated guidelines for reporting parallel group randomised trials. *J Clin Epidemiol* 2010; **63**: e1–37.

15 Liu GF, Lu K, Mogg R, Mallick M, Mehrotra DV. Should baseline be a covariate or dependent variable in analyses of change from baseline in clinical trials? *Stat Med* 2009; **28**: 2509–30.
